# Defective Intracortical Inhibition as a Marker of Impaired Neural Compensation in Amputees Undergoing Rehabilitation

**DOI:** 10.3390/biomedicines13051015

**Published:** 2025-04-22

**Authors:** Guilherme J. M. Lacerda, Lucas Camargo, Fernanda M. Q. Silva, Marta Imamura, Linamara R. Battistella, Felipe Fregni

**Affiliations:** 1 Instituto de Medicina Física e Reabilitação, Hospital das Clínicas HCFMUSP, Faculdade de Medicina, Universidade de São Paulo, São Paulo 04116-030, SP, Brazil; guilhermelacerda7@gmail.com (G.J.M.L.); marta.imamura@fm.usp.br (M.I.); linamara@usp.br (L.R.B.); 2 Neuromodulation Center, Center for Clinical Research Learning, Spaulding Rehabilitation Hospital and Massachusetts General Hospital, Harvard Medical School, Boston, MA 02115, USA; lcamargo@mgh.harvard.edu (L.C.); fmqsilva2006@gmail.com (F.M.Q.S.); 3 Departamento de Medicina Legal, Bioética, Medicina do Trabalho e Medicina Física e Reabilitação do da Faculdade de Medicina da Universidade de São Paulo (FMUSP), São Paulo 01246-903, SP, Brazil

**Keywords:** lower-limb amputation, neuroplasticity, neurophysiological biomarkers, transcranial magnetic stimulation

## Abstract

**Background/Objectives**: Lower-limb amputation (LLA) leads to disability, impaired mobility, and reduced quality of life, affecting 1.6 million people in the USA. Post-amputation, motor cortex reorganization occurs, contributing to phantom limb pain (PLP). Transcranial magnetic stimulation (TMS) assesses changes in cortical excitability, helping to identify compensatory mechanisms. This study investigated the association between TMS metrics and clinical and neurophysiological outcomes in LLA patients. **Methods**: A cross-sectional analysis of the DEFINE cohort, with 59 participants, was carried out. TMS metrics included resting motor threshold (rMT), motor-evoked potential (MEP) amplitude, short intracortical inhibition (SICI), and intracortical facilitation (ICF). **Results**: Multivariate analysis revealed increased ICF and rMT in the affected hemisphere of PLP patients, while SICI was reduced with the presence of PLP. A positive correlation between SICI and EEG theta oscillations in the frontal, central, and parietal regions suggested compensatory mechanisms in the unaffected hemisphere. Increased MEP was associated with reduced functional independence. **Conclusions**: SICI appears to be a key factor linked to the presence of PLP, but not its intensity. Reduced SICI may indicate impaired cortical compensation, contributing to PLP. Other neural mechanisms, including central sensitization and altered thalamocortical connectivity, may influence PLP’s severity. Our findings align with those of prior studies, reinforcing low SICI as a marker of maladaptive neuroplasticity in amputation-related pain. Additionally, longer amputation duration was associated with disrupted SICI, suggesting an impact of long-term plasticity changes.

## 1. Introduction

Amputation results in severe disability, impairing mobility, function, and independence, and reducing the quality of life, while also burdening healthcare systems [1]. The incidence of lower-limb amputation (LLA) is rising, ranging from 5.8 to 31 per 100,000 globally [2], with 1.6 million people affected in the USA [3]. Despite advances in prosthetics and rehabilitation, LLA patients face long-term functional limitations, disabilities, psychological challenges, and pain [4,5]. This highlights the need for practical, personalized rehabilitation strategies, as LLA rehabilitation is complex, owing to individual variability in patient profiles and amputation factors [6]. After amputation, connections between the peripheral and central nervous systems must be reorganized, inducing motor cortex reorganization [7,8]. The process of cortical reorganization can be related to enhanced sensitivity and control in the remaining limbs, but it also contributes to the development of phantom limb pain (PLP)—a painful sensation perceived in the region of a limb that has been amputated [9]—and phantom limb sensation (PLS), which refers to non-painful sensory perceptions in the missing limb, including feelings of movement, position, temperature, or general awareness of the limb [10], as discussed previously in neuroimaging and neurophysiological studies investigating potential biomarkers for these conditions [11,12,13]. In this context, transcranial magnetic stimulation (TMS) metrics may help understand compensatory mechanisms through interhemispheric changes in cortical excitability parameters in response to LLA. Understanding these mechanisms is critical to optimizing and individualizing amputee patients’ rehabilitation and treatment responses for pain management using non-invasive brain stimulation [11,14,15]. Therefore, TMS has emerged as a promising tool for assessing motor cortex excitability and intracortical inhibition in amputees [16].

TMS studies report increased motor cortex excitability and reduced intracortical inhibition in amputees [8,17,18]. In our previous systematic review [11], we observed that reduced inhibition may contribute to PLP, because downregulation mechanisms related to gamma-aminobutyric acid (GABA)-inhibitory circuits are related to cortical reorganization [19]. In addition, asymmetrical motor cortex reorganization has been linked to the timing of amputation, although not necessarily to PLP’s intensity [12]. At the same time, increased corticospinal excitability has been noted in trans-radial amputees, but not in trans-humeral cases [16]. Another systematic review [20] reported reduced intracortical inhibition in the affected hemisphere of amputee patients, but its role in PLP remains unclear.

In contrast, our previous study [21] found increased intracortical facilitation (ICF) in the affected hemisphere of patients with PLP. Similarly, Kleeva et al. [22] demonstrated that cortical excitation and inhibition disruption are linked to neuropathic pain, including PLP. Their study also connected electroencephalogram (EEG)-wave dysrhythmia—specifically, increased theta-band activity—to stable and fluctuating PLP. ICF and SICI can reflect the facilitatory and inhibitory interactions between interneurons in the motor cortex related to GABA and NMDA receptors [20,21,22,23]. A previous study observed that increased EEG theta power—a low-frequency band—was correlated with PLP intensity [24], but the results for EEG alpha and beta waves remained mixed. EEG oscillations demonstrate potential application as a neurophysiological biomarker, reflecting brain activity during a resting state or cognitive tasks [22,23,24,25]. Our previous studies observed that low-frequency bands, such as theta oscillations, were associated with compensatory mechanisms in patients with amputations, stroke, and chronic pain conditions [22,23,24,25]. These findings suggest that theta oscillations may enhance neuroplasticity and facilitate cortical reorganization, potentially serving as compensatory mechanisms for maladaptive changes in other cortical regions.

Although many studies have investigated the applicability of TMS parameters and other neurophysiological assessments to evaluate brain plasticity and mechanisms of adaptation after limb loss, there are still many gaps in the literature. Discrepancies persist owing to variations in study design, patient selection, and time since amputation, complicating the interpretation of potential TMS biomarkers [16]. Consequently, the precise role of cortical reorganization mechanisms and key TMS metrics reflected by inhibitory and excitatory cortical networks remains unclear. Therefore, we performed an exploratory cross-sectional study to investigate TMS metrics and their relationship with clinical and neurophysiological outcomes in patients with LLA. We hypothesized that increased corticospinal excitability in the affected hemisphere and increased facilitation in the unaffected hemisphere would be correlated with PLP, depression, and cognitive outcomes. Potentially, a more significant compensatory mechanism in the unaffected hemisphere could be expected to counterbalance the cortical disorganization on the affected side.

## 2. Materials and Methods

### 2.1. Study Design and Participants

This exploratory cross-sectional study analyzed baseline data from 59 participants in the cohort study “Deficit of Inhibition as a Marker of Neuroplasticity (DEFINE Study) in Rehabilitation: A Longitudinal Cohort Study Protocol” [26]. All of the participants provided written informed consent, and the study’s protocol was approved by the Ethics Committee for Research Protocol Analysis at the Hospital das Clínicas, Faculdade de Medicina, Universidade de São Paulo. All of the procedures were performed in accordance with the Declaration of Helsinki [27].

### 2.2. Eligibility Criteria

Participants were eligible for inclusion if they were aged 18 years or older, with a confirmed clinical diagnosis of lower-limb amputation, able to provide consent, clinically stable (for instance, controlled diabetes and hypertension) as determined by medical evaluation, and met the criteria for admission to the IMREA rehabilitation program. Individuals were excluded if they had any clinical or social condition that might interfere with their ability to participate in the rehabilitation program. The exclusion criteria were as follows: patients under 18, pregnancy, and bilateral LLA.

### 2.3. Sociodemographic, Clinical, and Other Neurophysiological Variables

A trained physician evaluated each participant’s eligibility by reviewing their medical history, conducting a physical examination, and collecting sociodemographic, clinical, and functional data. Sociodemographic data were collected from the participants, including age, biological sex, race, marital status, education level, employment status, body mass index (BMI), smoking, and alcohol consumption history. Clinical data included the amputation side, level, etiology, presence of phantom limb pain, phantom limb sensation, and residual limb pain. Participants were also assessed using validated instruments, such as the Hospital Anxiety and Depression Scale (HADS) [28], Epworth Sleepiness Scale (ESS) [29], Montreal Cognitive Assessment (MoCA) [30], and Functional Independence Measure (FIM) [31]. Additionally, we included the 5 min eyes-closed resting-state EEG variables in this analysis, using a 64-channel ANT Neuro EEG system (ANT Neuro, Enschede, the Netherlands). We followed the same EEG data collection and preprocessing methods described in our previous study [24].

### 2.4. Transcranial Magnetic Stimulation (TMS)

TMS assessments were conducted using a Magstim Rapid^®^ stimulator with a 70 mm figure-of-eight coil (Magstim Company Ltd., Whitland, Wales, UK). The coil was placed tangentially to the skull at a 45-degree angle to the sagittal line. The motor cortex of both hemispheres (right and left) was assessed, and the contralateral muscle responses were recorded using surface electromyography electrodes on the first dorsal interosseous muscle of the hand. The cortical area corresponding to the muscle was identified by marking 5 cm from the vertex (intersection of the nasion–inion lines and zygomatic arches) toward the ear tragus. The resting motor threshold (rMT) was defined as the minimum intensity at which a TMS pulse elicited a motor-evoked potential (MEP) of at least 50 µV peak-to-peak amplitude in 50% of the attempts. MEPs were then recorded at 130% of the rMT, with 10 measurements taken at 7 s intervals. The silent periods, short intracortical inhibition (SICI), and intracortical facilitation (ICF) were also measured. SICI was calculated as the ratio of MEP amplitude after the paired pulse (2 ms interval) to the baseline MEP, while ICF was determined using a 10 ms interval [32,33].

### 2.5. Statistical Analysis

We conducted a descriptive analysis of the demographic, clinical, functional, and neurophysiological data. Continuous variables were summarized using the median and interquartile range (IQR)—between the 25th and 75th percentiles—based on the central limit theorem. Categorical variables were summarized as frequencies and percentages. TMS metrics were considered to be dependent variables in this analysis, while other neurophysiological, functional, clinical, and sociodemographic variables were treated as independent variables. We evaluated the linearity assumption by visually inspecting the scatterplots for each independent variable, using a superimposed regression line.

TMS-dependent variables were categorized according to the amputation side in the “affected hemisphere” and “non-affected hemisphere” metrics. Next, we performed univariate linear regression analysis to identify the associations between the independent and TMS-dependent variables. Variables with a *p*-value < 0.20 in the univariate analysis were selected for inclusion in a preliminary multivariate analysis. To develop our final multivariate models, we employed a multi-criteria approach for confounder identification based on (1) prior research supporting physiological plausibility, (2) changes in β coefficients greater than 10%, and (3) verification of model assumptions including homoscedasticity, independence, and normality, as outlined by Osborne and Waters [34]. Additionally, we examined the relationships between the primary predictor variables and demographic or clinical factors, incorporating them into the final models where relevant. Confounders were identified based on their impact (greater than 20% change) on the regression coefficients from the unadjusted model. The results are reported as β coefficients with 95% CIs, *p*-values, variance inflation factors (VIFs), and model fits (adjusted R-squared), and missing data are reported in Table 1. Post-estimation checks were performed to verify the assumptions of linearity, normality, and homoscedasticity of the residuals, and multicollinearity was assessed using the variance inflation factor. Statistical significance was set at *p* < 0.05, and all analyses were conducted using Stata^®^ version 17.0 (StataCorp LLC, College Station, TX, USA), with α = 0.05.

Given the exploratory nature of this study, the regression analyses focused on identifying associations between the independent and dependent variables without predicting the impact of the independent variables on TMS values. Our findings should be interpreted as exploratory correlation tests, with TMS metrics examined for associations with clinical, sociodemographic, functional, and other neurophysiological characteristics.

## 3. Results

**Figure 1:**
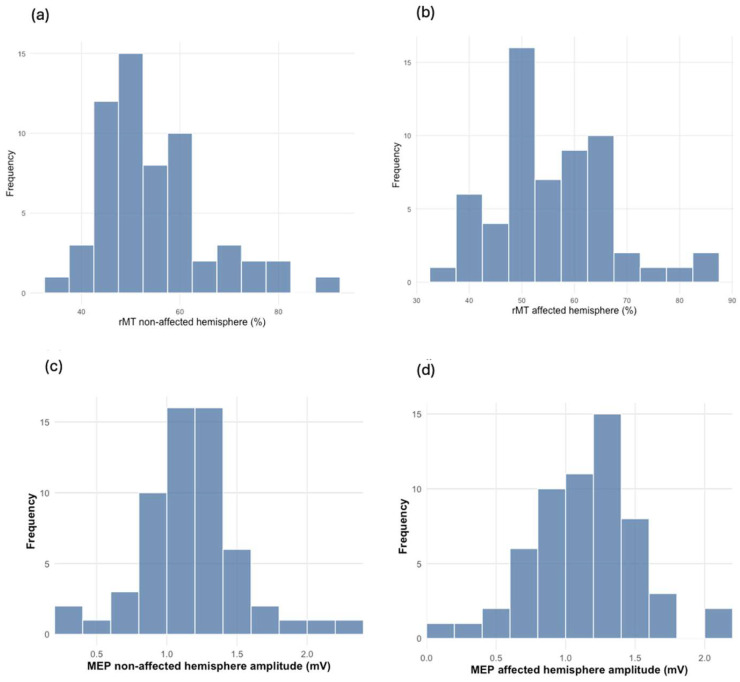
The distribution (n = 59) of rMT and MEP between the non-affected and affected brain hemispheres: (**a**) rMT distribution in the non-affected hemisphere (%); (**b**) rMT distribution in the affected hemisphere (%); (**c**) MEP distribution in the non-affected hemisphere (mV); (**d**) MEP distribution in the affected hemisphere (mV).

**Figure 2:**
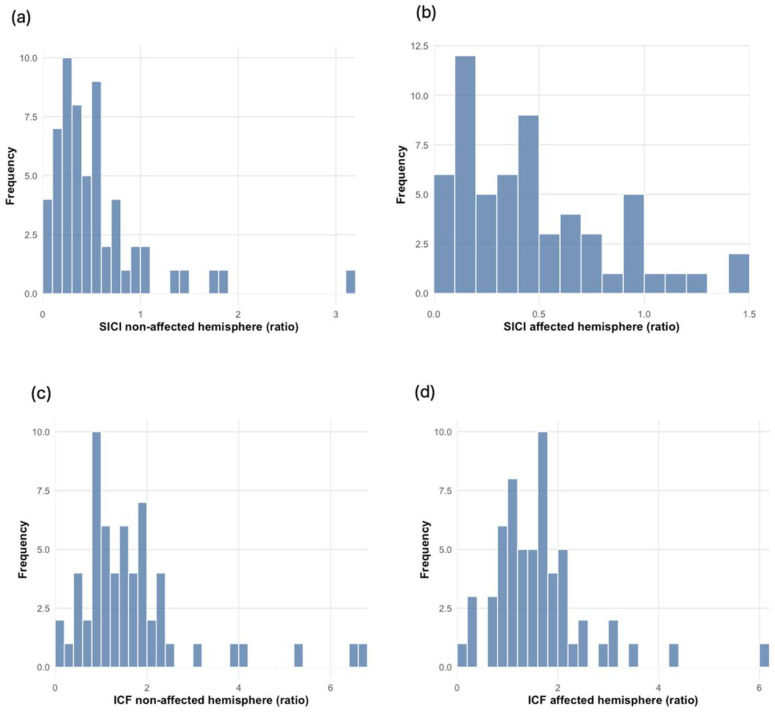
The distribution (n = 59) of SICI and ICF between the non-affected and affected brain hemispheres: (**a**) SICI distribution in the non-affected hemisphere (ratio); (**b**) SICI distribution in the affected hemisphere (ratio); (**c**) ICF distribution in the non-affected hemisphere (ratio); (**d**) ICF distribution in the affected hemisphere (ratio).

The median age was 47.0 years (IQR = 31–61). Most of the participants were male (84.75%). There were slightly more non-traumatic amputations (52.54%) than traumatic ones (47.46%). Left-sided amputations were more common (62.71%), and most of the amputations occurred above the knee (62.71%). PLP was homogeneously distributed in our study population (50%). A detailed description of the sociodemographic and clinical characteristics of the participants is provided in Table 1. Table 2 shows the medians and IQRs for the TMS parameters (rMT, MEPs, SICI, and ICF), and Figure 1 and Figure 2 display the data distribution. These are divided by hemisphere: the “affected hemisphere” corresponds to the side of the brain matching the amputated limb, while the “non-affected hemisphere” corresponds to the remaining limb. For instance, a patient with left LLA is considered to have an affected right hemisphere. Of the 59 participants, 37 underwent resting-state EEG recordings. The relative power of theta oscillations is presented in Table 3.

### 3.1. Regression Models

#### 3.1.1. Resting Motor Threshold (rMT) Models

Univariate analysis demonstrated a negative relationship between rMT in the non-affected hemisphere and PLP (b-coef. = 4.069, 95% CI [1.743, 9.881], *p* = 0.166). After the inclusion of other variables related to cognition (MoCA), functional status (FIM), and age, our multivariate model revealed a more substantial and statistically significant negative relationship between the rMT in the non-affected hemisphere and PLP (b-coef. = 7.453, 95% CI [0.753, 14.154], *p* = 0.030). In addition, the MoCA showed a significantly negative association (b-coef. = −1.181, 95% CI [−2.202, −0.160], *p* = 0.025). The model explained approximately 31% of the variance, with an R-squared of 0.3107, as shown in Table 4. No statistically significant differences were observed on the affected side.

#### 3.1.2. Motor-Evoked Potential (MEP) Models

The univariate analysis with the MEP in the affected hemisphere and the FIM showed a non-significant negative relationship (b-coef. = −0.023, 95% CI [−0.050, 0.004], *p* = 0.093). However, the multivariate model revealed a significant negative relationship between the MEP in the affected hemisphere and the FIM (b-coef. = −0.026, 95% CI [−0.051, −0.001], *p* = 0.044), as well as a positive correlation with sleep disorders (ESS) (b-coef. = 0.026, 95% CI [0.003, −0.049], *p* = 0.030). The model explained approximately 34% of the variance, with an R-squared of 0.344.

MEP in the non-affected hemisphere and the FIM also showed a significant negative relationship (b-coef. = −0.024, 95% CI [−0.049, 0.006], *p* = 0.056). Similar to the affected hemisphere, this relationship was statistically significant in our multivariate model (b = coef. = −0.034, 95% CI [−0.059, −0.009], *p* = 0.009) after including variables such as PLP, race, and biological sex. The model explained approximately 23% of the variance, with an R-squared of 0.228. A higher MEP demonstrated a positive relationship with race (non-White) in both hemispheres (affected and non-affected). Additional details are provided in Table 5.

#### 3.1.3. Short Intracortical Inhibition (SICI) Models

**Figure 3:**
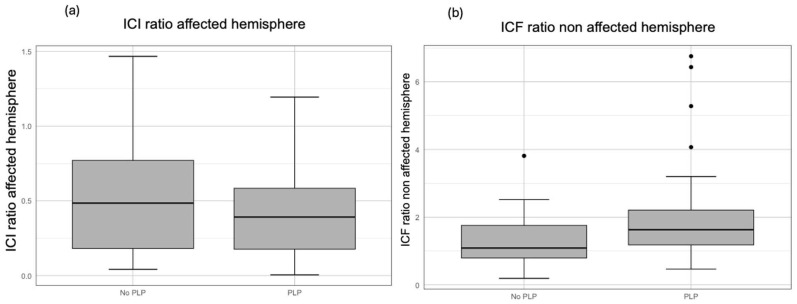
Boxplots comparing (**a**) ICI ratio in the affected hemisphere and the presence or absence of PLP (n = 58), and (**b**) ICF ratio in the non-affected hemisphere and the presence or absence of PLP (n = 49).

The univariate regression with SCI in the affected hemisphere and PLP showed a non-significant negative correlation (b-coef. = −0.134, 95% CI [−0.321, 0.054], *p* = 0.160). After the inclusion of the cause of amputation (traumatic or non-traumatic), race, biological sex, and age, our multivariate analysis showed a significant negative relationship between ICI in the affected hemisphere and the presence of PLP (b-coef. = −0.181, 95% CI = [−0.351, −0.011], *p* = 0.038). The model explained approximately 29% of the variance, with an R-squared of 0.291. However, this correlation was not significant in the contralateral hemisphere (unaffected hemisphere). A visual representation comparing SICI and PLP is displayed in Figure 3.

**Figure 4:**
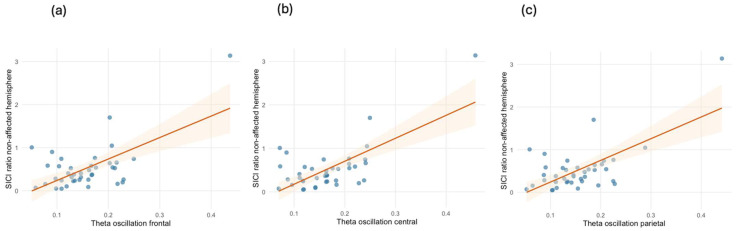
Scatterplots with fitted linear regression lines and 95% CIs (shaded band) displaying the positive associations between the SICI ratio in the non-affected hemisphere and theta oscillations in the (**a**) frontal, (**b**) central, and (**c**) parietal regions; n = 37.

Interestingly, in our univariate analysis, we observed a significant and positive association between SICI in the non-affected hemisphere and theta oscillations in the frontal lobe (b-coef. = 5.300, 95% CI = [3.403, 7.198], *p* < 0.001), as well as the central (b-coef. = 4.996, 95% CI = [2.863, 7.129], *p* < 0.001) and parietal regions (b-coef. = 5.075, 95% CI = [3.087, 7.063], *p* < 0.001) (see Figure 4). Even after adjusting for biological sex and age, these positive associations between SICI in the non-affected hemisphere and theta oscillations in the frontal, central, and parietal regions remained statistically significant in all areas, with R-squared values of 0.434, 0.500, and 0.460, respectively. No statistically significant association was observed between the SICI in the affected hemisphere and theta oscillations or the other EEG bands. The details of the multivariate models for SICI are presented in Table 6.

#### 3.1.4. Intracortical Facilitation (ICF) Models

Univariate regression analysis showed a significant positive correlation between ICF in the non-affected hemisphere and depression symptoms (HDS) (b-coef. = 0.191, 95% CI [0.054, 0.328], *p* = 0.007) (see Figure 4). This relationship remained statistically significant was slightly stronger in the multivariate model and (b-coef. = 0.204, 95% CI [0.077, 0.338], *p* = 0.002). The model also showed that race (non-White) was associated with reduced ICF (b-coef. = −1.019, 95% CI [−1.750, −0.288], *p* = 0.007), and it explained approximately 33% of the variance (R^2^ = 0.326). Regarding phantom limb pain (PLP), there was a significant positive association with ICF in the non-affected hemisphere (b-coef. = 0.815, 95% CI [−0.153, 1.478], *p* = 0.017). After adjusting for age, biological sex, race, and amputation etiology, the negative association remained significant (b-coef. = 0.662, 95% CI [1.298, 0.025], *p* = 0.042). This model explained 25% of the variance (R^2^ = 0.254).

The multivariate models for ICF are presented in Table 7. No significant associations were found for the ICF in the affected hemisphere.

A summary of our regression analysis between TMS parameters, EEG, and clinical variables is presented in Table 8.

## 4. Discussion

This study investigated the relationships between cortical excitability, PLP, and clinical and neurophysiological variables in individuals with limb amputation. First, individuals with PLP had increased ICF values, indicating a potential relationship between PLP and increased cortical excitability. In addition, ICF in the unaffected hemisphere, where we identified a positive association with depression, suggested that increased cortical facilitation is linked to higher depressive symptoms. Secondly, individuals with PLP had reduced SICI ratios in the affected hemisphere, indicating reduced inhibitory tone in the presence of PLP. Interestingly, SICI in the unaffected hemisphere was positively associated with EEG theta oscillations in the frontal, central, and parietal regions, indicating a link between inhibitory control and compensatory low-frequency oscillation activity in the unaffected hemisphere. The third significant finding involved the motor threshold in the unaffected hemisphere. Individuals without PLP had higher rMT amplitudes, suggesting reduced corticospinal excitability. Additionally, rMT was negatively associated with MoCA scores, indicating that better cognitive performance was linked to lower motor threshold values. The MEP amplitudes in the affected hemisphere were positively associated with sleepiness, as measured using the Epworth Sleepiness Scale [29], suggesting that greater corticospinal excitability may be linked to increased daytime sleepiness.

### 4.1. ICF in the Non-Affected Hemisphere

Our findings revealed that individuals with PLP had increased ICF values, indicating maladaptive cortical reorganization and an altered excitatory–inhibitory balance in the motor cortex. This maladaptive neuroplasticity in individuals experiencing PLP is probably driven by N-methyl-D-aspartate (NMDA)-receptor-mediated mechanisms [35,36]. In addition, we observed that the ICF in the unaffected hemisphere was positively associated with depression, suggesting that higher cortical facilitation is linked to higher depressive symptoms in individuals with limb amputation. This finding aligns with previous research, indicating that increased ICF is a characteristic neurophysiological marker of depressive symptoms. A systematic review and meta-analysis by Kinjo et al. [35] confirmed that patients with major depressive disorder exhibit heightened ICF compared with healthy controls, supporting the hypothesis that cortical excitatory–inhibitory imbalance plays a crucial role in mood disorders. The relationship between increased cortical excitability and depression may be mediated by altered neuroplasticity and impaired inhibitory control in the motor and prefrontal networks. Depressive symptoms have been linked to disruptions in GABAergic inhibition and excessive glutamatergic activity, contributing to both emotional dysregulation and motor system hyperexcitability [19,37]. In amputees, these alterations may be exacerbated by pain-related plasticity, reinforcing the link between neurophysiological dysfunction and psychological symptoms. The observed overlap between heightened ICF in PLP and depression suggests that both conditions may share common pathophysiological mechanisms, particularly involving excitatory–inhibitory imbalances in the motor and limbic circuits. Understanding these shared pathways may provide a foundation for developing targeted neuromodulation interventions aimed at restoring cortical balance and improving pain and mood outcomes in amputees.

### 4.2. SICI in Both Hemispheres

Our second key finding was that individuals without PLP had reduced SICI ratios in the affected hemisphere, suggesting a reduced inhibitory tone compared to those with PLP. Additionally, SICI in the unaffected hemisphere was positively associated with EEG theta oscillations in the frontal, central, and parietal regions, indicating a link between inhibitory control and large-scale oscillatory activity. Furthermore, the amputation duration was negatively associated with SICI in the unaffected hemisphere, suggesting that a longer duration since amputation may lead to reduced inhibitory function and cortical reorganization.

These findings align with prior evidence indicating that amputation is associated with altered inhibitory control in the motor cortex. A systematic review by Candido et al. [20] found that most studies supported the notion that PLP is associated with a state of cortical disinhibition. This review also highlighted significant heterogeneity across studies, with inconsistent associations between decreased SICI and clinical parameters, such as the presence or intensity of PLP. Although it remains unclear whether reduced SICI directly contributes to PLP, these results emphasize the role of inhibitory dysfunction in post-amputation neuroplasticity.

One crucial finding in this study is that we demonstrated that SICI is a critical variable correlated with the presence of PLP, but not with its intensity. This distinction suggests that, while reduced ICI may contribute to the development of PLP, other neural mechanisms likely regulate the severity of the pain experience. Interestingly, our results align with findings from a previous study [12], where SICI, ICF, and cortical mapping were not found to be directly associated with PLP. These findings indicate that low SICI could be a marker of impaired cortical compensation, potentially increasing susceptibility to PLP. However, once PLP is established, its intensity may be modulated by distinct neural circuits, such as those involved in central sensitization, maladaptive plasticity, and altered thalamocortical connectivity.

The association between SICI in the unaffected hemisphere and EEG theta oscillations further suggests that inhibitory control mechanisms are not restricted to the affected hemisphere but may influence broader cortical networks. Theta oscillations play key roles in sensory integration, attention, and inhibitory processing [24,25]. Their positive association with ICI suggests a compensatory mechanism in which preserved inhibitory function supports large-scale cortical stability [33,38,39]. Previous research in stroke patients has highlighted the role of theta oscillations in neuroplastic adaptation, where shifts in oscillatory activity reflect ongoing cortical reorganization and recovery [33]. While the mechanisms of cortical adaptation differ between stroke and amputation, the presence of theta-oscillatory activity concerning inhibitory control in amputees may similarly indicate an adaptive neural response aimed at stabilizing network function. Meanwhile, the observed negative relationship between amputation duration and SICI in the unaffected hemisphere may indicate a progressive decline in inhibitory control over time, potentially contributing to maladaptive plasticity and prolonged pain.

As discussed in previous studies, pain sensitivity evolves in a time-dependent manner following amputation, exhibiting an inverted U-shaped relationship with time since amputation [40]. While early post-amputation adaptations may involve peripheral healing and functional reorganization, longer durations appear to be associated with increased pain sensitivity, potentially due to the development of chronic pain conditions, such as phantom limb pain or central sensitization [41,42]. These findings suggest that prolonged post-amputation changes in cortical excitability and inhibitory control may be key factors in shaping long-term pain outcomes in amputees. Future studies should explore whether interventions aimed at preserving intracortical inhibition over time can mitigate maladaptive plasticity and reduce the risk of chronic pain following amputation. From a clinical perspective, these findings highlight the importance of considering inhibitory network dysfunction when evaluating pain and neural reorganization after limb amputation. Interventions aimed at restoring intracortical inhibition, such as non-invasive brain stimulation techniques targeting the affected hemisphere, may hold promise for managing PLP.

### 4.3. rMT in the Non-Affected Hemisphere

Our third significant finding involves the motor threshold in the non-affected hemisphere. Individuals without PLP had lower motor threshold amplitude values, suggesting increased cortical excitability. This result aligns with our first finding, where individuals with PLP exhibited higher ICF, reinforcing the idea that altered cortical excitability may differentiate those with and without PLP. These findings are consistent with previous research indicating that cortical excitability imbalances play a role in pain modulation [35]. Additionally, we found a negative association between motor threshold and MoCA scores, suggesting that better cognitive performance was linked to lower motor threshold values. Our findings align with those of previous research demonstrating a relationship between cortical excitability and cognitive function. Specifically, a meta-analysis on cortical excitability in Alzheimer’s disease and mild cognitive impairment found that individuals with these conditions exhibited increased cortical excitability, which may reflect compensatory mechanisms or a pathological imbalance in excitatory and inhibitory networks [43]. The study suggested that hyperexcitability is linked to cognitive dysfunction, possibly due to the degeneration of inhibitory GABAergic interneurons and alterations in cholinergic modulation, leading to a loss of inhibitory control over cortical activity [18,43]. The observed negative association between motor threshold and MoCA scores in our study suggests that increased cortical excitability may serve as a compensatory mechanism supporting cognitive function. This relationship underscores the interplay between motor and cognitive systems, which may be mediated by shared neural circuits and neurotransmitter systems. Understanding these interactions could inform therapeutic strategies, particularly those involving non-invasive brain stimulation techniques, to optimize cognitive and motor function in this population.

### 4.4. MEP Amplitudes in the Affected Hemisphere

Furthermore, we observed that the MEP amplitudes in the affected hemisphere were positively associated with sleepiness, as measured by the ESS. This finding suggests that greater corticospinal excitability may be linked to increased daytime sleepiness. Previous research has explored the relationship between cortical excitability and sleepiness [44]. For instance, a study investigated cortical excitability signatures corresponding to varying degrees of sleepiness in humans. The researchers found that changes in cortical excitability, as measured by TMS, were reflective of the participants’ subjective sleepiness levels. Specifically, increased sleepiness was associated with alterations in both excitatory and inhibitory synaptic activity, indicating a complex interplay between cortical excitability and sleep propensity [44]. Additionally, studies on patients with obstructive sleep apnea syndrome (OSAS) have demonstrated altered cortical excitability associated with excessive daytime sleepiness. In fact, Antonello et al. [45] utilized TMS to assess cortical excitability in OSAS patients and found significant correlations between increased cortical excitability and higher ESS scores. These findings suggest that sleep disorders characterized by excessive daytime sleepiness may involve dysregulation of cortical excitability. The observed association between increased MEP amplitudes and higher ESS scores in our study aligns with these previous findings, indicating that heightened corticospinal excitability may be a neurophysiological marker of increased daytime sleepiness. This relationship could be mediated by several mechanisms, including disruptions in the balance of excitatory and inhibitory neurotransmission, alterations in synaptic plasticity, or compensatory responses to sleep deprivation.

## 5. Conclusions

Despite the limitations of a cross-sectional study design and small sample size, we observed significant differences in TMS variables between the affected and non-affected brain hemispheres in patients with LLA. The presence of PLP was found to be related to an increase in ICF, rMT, and SICI, reflecting an altered inhibitory control in the motor cortex in this population. Also, the significant positive association between theta oscillations in different regions and SICI demonstrates compensatory neurophysiological mechanisms in the non-affected hemisphere after a contralateral amputation. Additionally, MEP seems to be a potential biomarker of sleep disorders and functional recovery. These findings support the potential use of TMS as a biomarker for medical rehabilitation and brain plasticity, providing essential insights into the association between cortical excitability and the presence of PLP, which could be applied in the future for diagnosis or treatment response. Therefore, longitudinal studies with larger sample sizes could provide deeper insights into the temporal dynamics of cortical reorganization, clarify long-term neurophysiological changes and clinical outcomes, and help identify predictive biomarkers to improve personalized rehabilitation interventions and quality of life for individuals with LLA.

## Figures and Tables

**Table 1:** Sociodemographic and clinical features of the 59 lower-limb amputation (LLA) patients included in this study.

Demographic and Clinical Variables	Median (IQR) or n (%)	Missing Data (n)
Age, mean (SD)	47.0 (31–61)	None
Biological sex		None
Male	50 (84.75)	
Female	9 (15.25)	
BMI (kg/m^2^), mean (SD)	25.15 (22.75–28.62)	None
Race		None
White	20 (33.90)	
Non-White	39 (66.10)	
Years of education, mean (SD)	9.51 (3.87)	None
Marital status		None
Single	23 (38.98)	
Married	24 (40.68)	
Divorced	10 (16.95)	
Widowed	2 (3.39)	
Amputation etiology		None
Traumatic	28 (47.46)	
Non-traumatic	31 (52.54)	
Amputation side		None
Right	22 (37.29)	
Left	37 (62.71)	
Amputation level, n (%)		
Above the knee	37 (62.71)	
Below the knee	22 (37.29)	
Hand dominance		None
Right	54 (91.53)	
Left	5 (8.47)	
Time since amputation	18.00 (11.30–30.33)	None
Epworth Sleepiness Scale (ESS)	8.00 (6.00–12.00)	3
Phantom limb pain (PLP)		1
yes	29 (50)	
no	29 (50)	
Phantom limb sensation (PLS)		1
yes	43 (74.14)	
no	15 (49)	
Residual limb pain (RLP)		2
yes	12 (21.05)	
no	45 (78.95)	
Hospital Anxiety Scale (HAS)	3.00 (1.50–6.00)	10
Hospital Depression Scale (HDS)	2.00 (0.00–4.00)	10
Functional Independence Measure (FIM)	116 (114–118)	10
Montreal Cognitive Assessment (MoCA)	23 (20–24)	12

**Table 2:** Transcranial magnetic stimulation (TMS) findings of the 59 patients with lower-limb amputation (LLA) included in this study.

Dependent Variables	Median (IQR)
Resting motor threshold (%)	
Non-affected hemisphere	54.0 (46.5–60)
Affected hemisphere	54.0 (49.0–63.0)
Motor-evoked potential (MEP), mV	
Non-affected hemisphere	1.14 (0.98–1.37)
Affected hemisphere	1.14 (0.95–1.36)
Short intracortical inhibition (SICI) ratio	
Non-affected hemisphere	0.41 (0.24–0.64)
Affected hemisphere	0.41 (0.17–0.66)
Intracortical facilitation (ICF) ratio	
Non-affected hemisphere	1.41 (0.93–1.95)
Affected hemisphere	1.51 (1.05–1.95)

**Table 3:** Electroencephalogram (EEG) theta oscillation relative power (%) findings of 37 patients with lower-limb amputation (LLA) were included in this study.

Theta Oscillations	Median (IQR)
Frontal	14.66 (10.90–20.26)
Central	16.33 (11.78–20.85)
Parietal	14.57 (11.05–18.78)

**Table 4:** Resting motor threshold (rMT) multivariate model.

rMT, Non-Affected Hemisphere, n = 58	Beta Coefficient	95% CI	*p*-Value	VIF	R-Squared
PLP	7.453	[0.753, 14.154]	0.030	1.125	0.311
MoCA	−1.181	[−2.202, −0.160]	0.025	1.050	
FIM	0.740	[−0.094, 1.573]	0.080	1.144	
Age	0.173	[−0.013, 0.360]	0.068	1.063	

Phantom limb pain (PLP); Montreal Cognitive Assessment (MoCA); Functional Independence Measure (FIM).

**Table 5:** Motor-evoked potential (MEP) multivariate model.

MEP, Affected Hemisphere, n = 46	**Beta Coefficient**	**95% CI**	***p*-Value**	**VIF**	**R-Squared**
FIM	−0.026	[−0.051, −0.001]	0.044	1.057	0.344
ESS	0.026	[0.003, 0.049]	0.030	1.069	
Race	0.343	[0.123, 0.564]	0.003	1.089	
Biological sex	−0.270	[−0.558, 0.009]	0.058	1.080	
MEP, Non-Affected Hemisphere, n = 48					
FIM	−0.034	[−0.059, −0.009]	0.008	1.077	0.228
PLP	−0.188	[−0.403, 0.027]	0.085	1.186	
Race	0.185	[−0.031, 0.400]	0.003	1.137	
Biological sex	−0.091	[−0.377, 0.195]	0.058	1.167	

Functional Independence Measure (FIM); Epworth Sleepiness Scale (ESS); phantom limb pain (PLP).

**Table 6:** Short intracortical inhibition (SICI) multivariate model.

SICI, Affected Hemisphere, n = 58	**Beta Coefficient**	**95% CI**	***p*-Value**	**VIF**	**R-Squared**
PLP	−0.181	[−0.011, −0.351]	0.038	1.034	0.291
Etiology	0.297	[0.108, 0.485]	0.003	1.263	
Race	−0.197	[−0.375, −0.019]	0.030	1.022	
Biological sex	0.005	[−0.242, 0.251]	0.970	1.140	
Age	0.002	[−0.004, 0.007]	0.521	1.143	
SICI, Non-Affected Hemisphere, n = 37					
Theta, frontal	5.294	[3.119, 7.468]	<0.001	1.048	0.434
Biological sex	−0.463	[−0.838, 0.113]	0.130	1.029	
Age	0.001	[−0.008, 0.010]	0.796		
Theta, central	5.383	[3.449, 7.317]	<0.001	1.016	0.500
Biological sex	−0.257	[−0.699, 0.185]	0.246	1.006	
Age	0.004	[−0.007, 0.009]	0.915	1.013	
Theta, parietal	5.116	[3.146, 7.165]	<0.001	1.005	0.460
Biological sex	−0.254	[−0.713, 0.206]	0.269	1.006	
Age	−0.002	[−0.011, 0.006]	0.617	1.001	

**Table 7:** Intracortical facilitation (ICF) multivariate model.

ICF, Non-Affected Hemisphere, n = 49	Beta Coefficient	95% CI	*p*-Value	VIF	R-Squared
PLP	0.662	[0.025, 1.298]	0.042	1.034	0.254
Etiology	0.612	[−0.093, 1.317]	0.088	1.263	
Race	−0.700	[−1.365, −0.034]	0.040	1.022	
Age	0.009	[−0.011, 0.029]	0.386	1.143	
Biological sex	0.292	[−0.631, 1.215]	0.529	1.140	
Depression (HDS)	0.204	[0.077, 0.338]	0.002	1.017	0.326
Race	−1.019	[−1.750, −0.288]	0.007	1.021	
Age	0.014	[−0.007, 0.036]	0.185	1.008	
Biological sex	0.706	[−0.294, 1.707]	0.162	1.009	

Phantom limb pain (PLP); Hospital Depression Scale (HDS).

**Table 8:** Summary of regression analysis between TMS parameters and other EEG and clinical variables.

TMS Parameters	Affected Hemisphere	Non-Affected Hemisphere
↑ **MT**		↑ PLP ↓ MoCA ↑ FIM
↑ **MEP**	↓ FIM	↓ FIM
	↑ ESS	
↑ **SICI**	↓ PLP	↑ Theta, frontal
		↑ Theta, central ↑ Theta, parietal
↑ **ICF**		↑ PLP ↑ Depression

↑↓: The direction of the correlation between the dependent (TMS) and independent (EEG and clinical) variables.

## Data Availability

The data that support the findings of this study are available from the corresponding author upon reasonable request, due to reasons of privacy.

## Abbreviations

The following abbreviations are used in this manuscript:

CI	Confidence interval
EEG	Electroencephalogram
ESS	Epworth Sleepiness Scale
FIM	Functional Independence Measure
HAS	Hospital Anxiety Scale
HDS	Hospital Depression Scale
ICF	Intracortical facilitation
LLA	Lower-limb amputation
MEP	Motor-evoked potential
MoCA	Montreal Cognitive Assessment
OSAS	Obstructive sleep apnea syndrome
PLP	Phantom limb pain
PLS	Phantom limb sensation
RLP	Residual limb pain
rMT	Resting motor threshold
SICI	Short intracortical inhibition
TMS	Transcranial magnetic stimulation
VIF	Variance inflation factor
